# Underreporting of Death by COVID-19 in Brazil's Second Most Populous State

**DOI:** 10.3389/fpubh.2020.578645

**Published:** 2020-12-15

**Authors:** Thiago Henrique Evangelista Alves, Tafarel Andrade de Souza, Samyla de Almeida Silva, Nayani Alves Ramos, Stefan Vilges de Oliveira

**Affiliations:** Department of Collective Health, Faculty of Medicine, Federal University of Uberlândia, Uberlândia, Brazil

**Keywords:** COVID-19 deaths, underreporting, SARI deaths, Minas Gerais, Brazil

## Abstract

The COVID-19 pandemic brings to light the reality of the Brazilian health system. The underreporting of COVID-19 deaths in the state of Minas Gerais (MG), where the second largest population of the country is concentrated, reveals government unpreparedness, as there is a low capacity of testing in the population, which prevents the real understanding of the general panorama of SARS-CoV-2 dissemination. The goals of this research are to analyze the causes of deaths in different Brazilian government databases (Civil Registry Transparency Portal and InfoGripe) and to assess whether there are sub-records showing an unexpected increase in the frequency of deaths from causes clinically similar to COVID-19. A descriptive and quantitative analysis of the number of deaths by COVID-19 and similar causes was performed in different databases. Our results demonstrate that different official sources had a discrepancy of 109.45% between these data referring to the same period. There was also a 758.57% increase in SARI deaths in 2020, when compared to the average of previous years. Finally, it was shown that there was an increase in the rate of pneumonia and respiratory insufficiency (RI) by 6.34 and 6.25%, respectively. In conclusion, there is an underreporting of COVID-19 deaths in MG due to the unexplained excess of deaths caused by SARI, respiratory insufficiency, and pneumonia compared to previous years.

## Introduction

Coronavirus 2 (CoV-2) is a new beta-coronavirus related to severe acute respiratory syndrome (SARS) that emerged in December 2019 in China and became a pandemic in March 2020 due to its high infection and mortality rates (1–3). COVID-19 was the official name given to the disease caused by the new coronavirus of 2019 (SARS-CoV-2) by the World Health Organization (WHO) (1).

The first epicenter of COVID-19 was observed in Wuhan, the capital of Hubei, China, in December 2019, based on several notifications of pneumonia cases (4).

Since then, COVID-19 has rapidly spread around the world and, as of May 12, 2020, more than 4.4 million cases of the disease have been confirmed, causing over 299,000 deaths worldwide (5). Of this total, Brazil registered its first case on February 26 and in May totaled more than 188,000 cases and more than 13,000 deaths (6, 7).

COVID-19 is classified according to symptom severity. Patients with the mild form (80% of the cases) present fever, dry cough, chills, malaise, muscle pain, and sore throat. Patients with the moderate form present fever, respiratory symptoms, and radiographic characteristics. Severe patients (5% of the cases) manifest dyspnea (> 30 bpm), low oxygen saturation (<93%), and a low PaO2/FiO2 ratio (<300 mmHg), which may evolve into respiratory failure, septic shock, and multiple organ failure (8–10).

Furthermore, increased age and the presence of comorbidities, such as hypertension, diabetes, and coronary disease, are associated with mortality in COVID-19 patients (11, 12).

The accurate diagnosis of COVID-19 is carried out by searching for the genetic material of the virus and, in a complementary way, by imaging methods. Computed tomography and radiographs can identify lesions in the lungs due to viral multiplication (13, 14).

Laboratory confirmation is essential for the timely management of cases to avoid the spread of transmission. However, the government of Brazil was unprepared (7, 15, 16), and is far below the ideal number of tests for COVID-19 (17) as there are not enough laboratory inputs to understand the overall panorama of the spread of the virus. Furthermore, confirmatory molecular tests depend on the availability of imported reagents, which are globally scarce, and on government investments that prioritize this strategy. This scenario has led to a delay in Brazil in the confirmation of the number of COVID-19 cases and deaths (18). These aspects become more aggravated when a patient dies, because effective testing for these cases is even more difficult. Considering the studies done to date, the recommendation is to collect blood and sputum to perform the culture, since these samples have a higher viral load (19).

The difficulty of death registration has also been presented in the state of Minas Gerais, which, by the end of April 2020, had 584 suspected death notifications, of which 81 (13%) had not yet been confirmed or discarded (20). Thus, it is possible to state that there is a disparity between the real number of COVID-19 deaths and the numbers that are reported in different Brazilian sources of information, since not all deaths have been confirmed or excluded and are potentially being caused by factors other than COVID-19.

The present study aims to analyze the causes of death in the notary records and in the Brazilian national disease notification system records, and thus evaluate the sub-registries and the possible increase in the frequency of deaths with causes clinically comparable with COVID-19 in the Minas Gerais territory.

## Methods

### Data Preparation

The state of Minas Gerais (MG) has an estimated population of 21,168,791 people in a territory of 586,521.121 km^2^, has the second largest population, and is the fourth largest state in the country (21). Its human development index (HDI) is 0.731, with a population composed of ~22.25% aged 0–15 years, 69.31% aged 15 to 64 years, and 8.12% aged over 65 years (22).

For this study, the death records from the Civil Registry Transparency Portal database (“https://transparencia.registrocivil.org.br/inicio”) (23) were analyzed from January to the first week of June 2020 (epidemiological weeks - EWs 1–23) in the state of MG according to their death cause. Additionally, to assess severe acute respiratory infection (SARI) excess deaths and COVID-19 deaths in these same EWs, information from the InfoGripe database (“http://info.gripe.fiocruz.br/”) (24) was accessed referring to the range of years 2017 to 2020.

The Civil Registry Transparency Portal is a free access platform developed to provide information about births, marriages, and deaths. Due to the COVID-19 pandemic, these data are being grouped in the special section “COVID Registral Panel” (“https://transparencia.registrocivil.org.br/especial-covid”) (25). The information presented here (accessed on 06/18/2020) is based on death certificates (DCs), presenting only one cause of death for each certificate (23). On DCs, the causes of death follow a specific order of completion and are named according to CID-10. Thus, the cause mentioned in the last line of the DC is considered the reason for the death to which it refers. With this data source, we also evaluated the excess deaths from causes that present clinical compatibility with COVID-19, according to the following etiology: pneumonia; respiratory insufficiency (RI), septicemia (sepsis/septic shock), and undetermined (causes of deaths linked to respiratory diseases, but not conclusive) (23).

The second data source, “InfoGripe database,” is a platform of the Oswaldo Cruz Foundation (Fiocruz) (24) that aims to monitor and present alert levels for reported cases of SARI. On this platform, the records of SARI and COVID-19 were selected on 06/18/2020 according to the first and 23rd epidemiological weeks (EWs) from 2017 to 2020 for the state of Minas Gerais.

It is important to note that obtaining the data from the Civil Registry Transparency Portal takes up to 14 days, from the notification from the family to the update of the platform. The data analyzed here were obtained through those already in the system, without considering future updates (25). Similarly, the approximate period between notification in SINAN and the data presented on the InfoGripe website is 22 days (26).

The data were collected and analyzed in a spreadsheet by descriptive statistics and presented in raw numbers, relative frequency, and central tendency measures. The mortality rate was calculated using population data per 100,000 inhabitants from the state of Minas Gerais. To assess the excess SARI deaths per EW, the average, minimum, and maximum values of deaths from the years 2017 to 2019 were calculated and compared with the pattern of distribution in the same period of 2020. To assess the deaths from causes clinically comparable with COVID-19 in 2020 the data from each set were normalized to create a heatmap. All graphs were prepared using the *GraphPadPrism 7* software (GraphPad Software, Inc. San Diego, CA).

### Statistics

Student's *t* test for independent samples was used to compare the average SARI deaths from 2017 to 2019 with the SARI deaths from 2020 during epidemiological weeks 1–23, using the *GraphPad Prism 7* software (GraphPad Software, Inc. San Diego, CA). A *p* value < 0.05 was considered statistically significant. Furthermore, it was not possible to obtain the data from the Civil Registry Transparency Portal for the years 2017 and 2018 for deaths by other respiratory causes clinically comparable with COVID-19, a fact that made the statistical test for that case impossible.

## Results

### Number of COVID-19 Deaths in the Databases

A total of 731 COVID-19 deaths were identified on the Civil Registry Transparency Portal, this number was 109.45% greater than the 349 total deaths registered in the InfoGripe database during the same period. While a total of 1,540 SARI deaths were identified on the InfoGripe database, this number was 539% greater than the 241 total deaths registered in the Civil Registry Transparency Portal during the same period (Table 1).

**Table 1:** Number of deaths by COVID-19 and SARI according to the information on the Civil Registry Transparency Portal and the InfoGripe database.

	**January**		**February**		**March**		**April**		**May**		**June^a^**		**Total**	**Mean (SD)**	**Deaths per 100,000 inhabitants**
**COVID-19 deaths**	***N***	**%**	***N***	**%**	***N***	**%**	***N***	**%**	***N***	**%**	***N***	**%**	***N***		
Civil Registry Transparency Portal	0	0	0	0	49	6.7	194	26.54	353	48.29	135	18.47	731	121.83 (136.98)	3.45
Infogripe database	0	0	1	0.29	48	13.75	94	26.94	163	46.7	43	12.32	349	58.17 (62.06)	1.65
**SARI deaths**															
Civil Registry Transparency Portal	8	3.32	6	2.49	35	14.52	84	34.85	88	36.51	20	8.30	241	40.17 (37.0)	1.14
Infogripe database	19	1.23	30	1.95	405	26.30	459	29.81	509	33.05	118	7.66	1540	256.67 (225.26)	7.27

a *Corresponding to the first week of June*.

### Excess Deaths From Causes Clinically Comparable With COVID-19 in 2020

**Figure 1:**
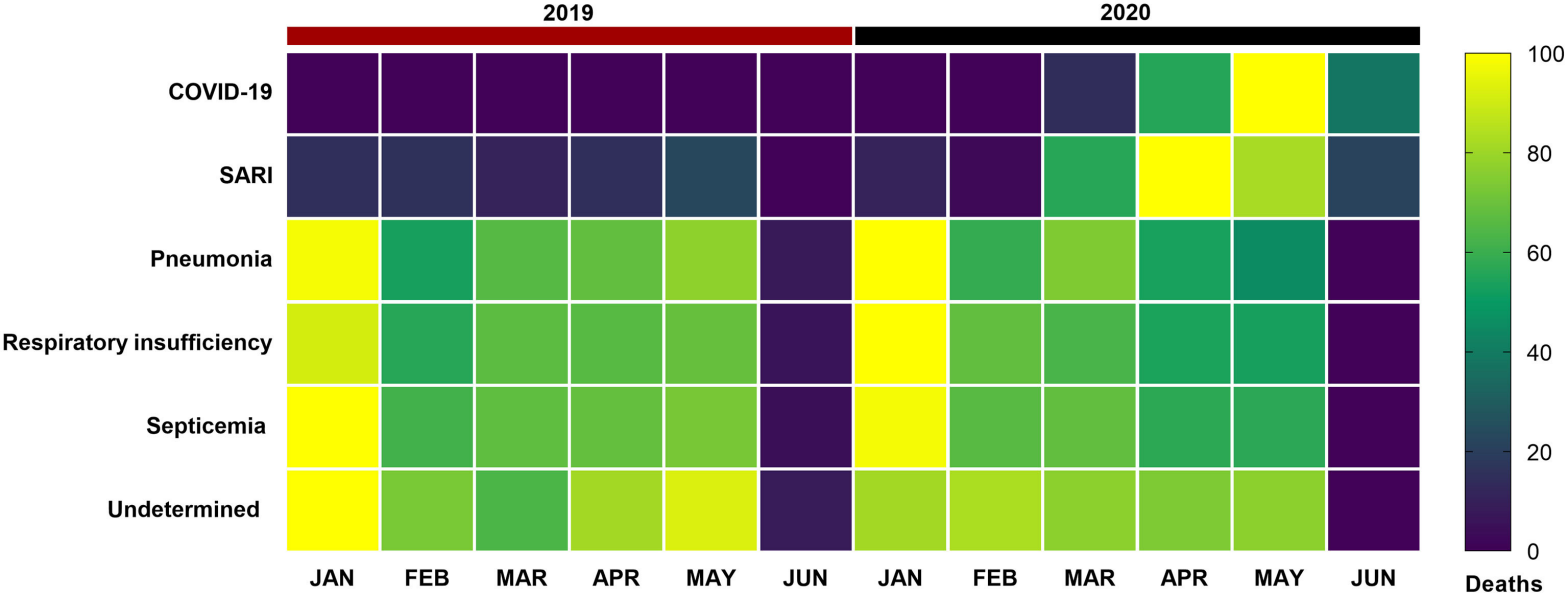
Distribution of deaths by COVID-19, severe acute respiratory infection (SARI), pneumonia, respiratory failure (RSI), sepsis, and undetermined causes (deaths related to respiratory diseases, but not conclusive), according to the Civil Registry Transparency Portal, from January to the first week of June 2019 and 2020 in the state of Minas Gerais, Brazil. Each rectangle represents the percentage of deaths by disease colored by its normalized intensity scale from blue (fewer deaths) to yellow (more deaths).

The evaluation of the causes of death on the Civil Registry Transparency Portal showed an increase in the frequency of SARI deaths in 2020 in relation to the number of deaths from the same cause in 2019 and regarding the rates of the other diseases within the same period, no increase was observed (Figure 1).

The increase of SARI deaths in 2020 was in the order of 312.98% compared to 2019 (**Supplementary Tables 1, 2**).

### Excess SARI Deaths in 2020

**Figure 2:**
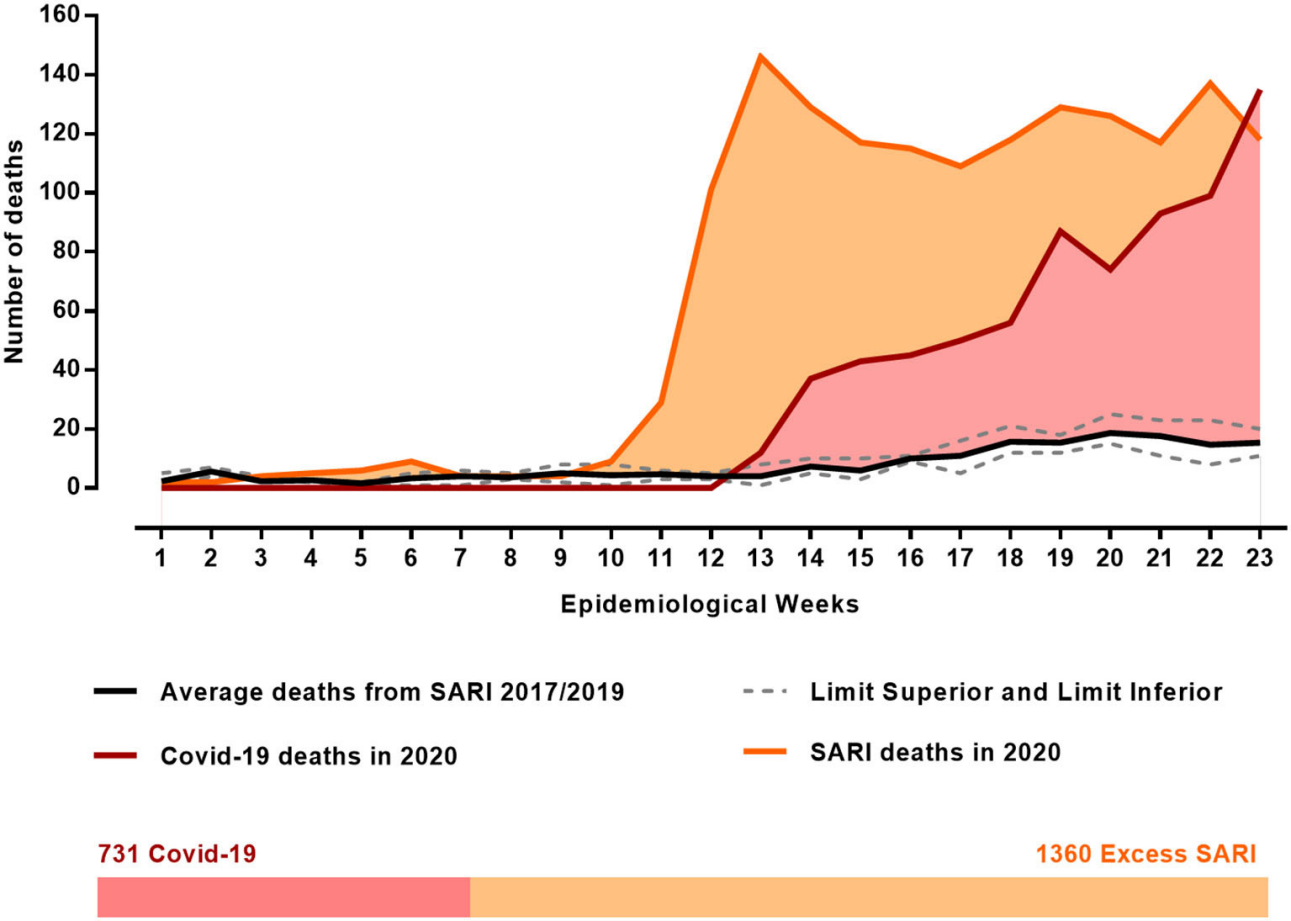
Evaluation of excess deaths from SARI in 2020, during epidemiological weeks 1–23 compared with the average deaths in the same weeks of the previous years (2017–2019), according to the InfoGripe database. Data on deaths by COVID-19 during the same epidemiological weeks of 2020 were obtained on the Civil Registry Transparency Portal database in the state of Minas Gerais, Brazil.

The analysis of the excess SARI deaths in 2020, according to epidemiological weeks 1–23, showed a significant increase in SARI deaths (66.95 ± 59.46) compared to the average of previous years 2017–2019 (7.79 ± 6.26) (*p* < 0.0001). As shown in Figure 2, the increase in SARI deaths was approximately 758.57%. Such a high rise in SARI deaths was observed in epidemiological week number 10 while the records of COVID-19 deaths in the state of MG were only reported from epidemiological week number 12 (**Supplementary Tables 3–6**).

## Discussion

The causes of death from respiratory diseases in Minas Gerais registered in the Civil Registry Transparency Portal and in the InfoGripe database were evaluated as proposed in our objective; however, we observed divergences between the different databases used. When analyzing deaths from causes clinically comparable with COVID-19, there was a significant increase in deaths from SARI in 2020 when compared to the averages of 2017, 2018, and 2019. As this increase occurred weeks before the registration of deaths by COVID-19 and it was an increase that exceeded the upper average of the previous 3 years, it is believed that deaths due to COVID-19 were underreported, since deaths from this cause may have been being registered as SARI.

The COVID-19 situation is particularly challenging because, besides being a new and unprecedented disease, it is also capable of triggering other conditions, such as pneumonia and SARI, which can be characterized as the main cause of death. In other words, COVID-19 may be the underlying cause; that is, it may not be the direct cause of death that has been registered. In this perspective, there is a subjectivity bias, since physicians can neither confirm nor deny that a death was caused by COVID-19 according to their clinical knowledge without the need of laboratory tests (27). This finding corroborates data from Hubei, China and Northern Italy, where mortality calculations were adjusted for the biases of preferential verification, symptomatic and severe cases, and the delay in death records. An increase in the mortality rate was found, which confirmed the underreporting of COVID-19 deaths in those regions (28). The context of the similarity of signs and symptoms between COVID-19 and SARI and of the subjectivity bias is real in Minas Gerais. This is reflected in the significant increase in deaths from SARI compared to the average of previous years at a time when COVID-19 occurred.

As this increase in SARI deaths in 2020 significantly exceeded the average number of deaths from this cause in 2017, 2018, and 2019 and due the current scenario of the COVID-19 pandemic, we believe this increase was, in fact, cases of COVID-19 registered as SARI, which would represent an underreporting of deaths by COVID-19. But still, in relation to underreporting in Brazil, the Ministry of Health (MH) reports that the number of underreported deaths is low according to the Mortality Information System (MIS), because states and municipalities are advised to include deaths from COVID-19, either confirmed cases or only suspected cases, in the system as a priority in order to advance analysis of these cases (29).

Another issue that should be analyzed is that, although the Civil Registry Transparency Portal takes into consideration both confirmed and suspected deaths, the MH only discloses laboratory-proven COVID-19 deaths in its reports (29). However, suspected deaths need to be considered in the count, even though it is noted that they have not been confirmed. This is stated because it is known that many of these deaths will not be able to be analyzed, given the difficulties in collecting, transporting, and wrapping the post-mortem samples. Thus, if they are not mentioned, there will be an underestimation of the real situation in Brazil and, consequently, in the state of MG.

The Brazilian Ministry of Health also highlights that on the same death certificate more than one cause of death can be described, so that the registration of COVID-19 can be associated with other diseases. However, the Civil Registry Transparency Portal presents these causes separately, even those included or registered on the same death certificate. Thus, for MH, it is not possible to add only the deaths made available on the portal by different diseases, as they would generate false overreporting. The solution would be a complete investigation that considers each death and the causes mentioned on the death certificate (29). However, according to the hierarchical criteria exposed in the Transparency Portal of the Civil Registry, only one cause of death is selected to make the count, and not all causes are presented on the same death certificate (25), which validates the exposed data on that platform and the information presented here.

It is worth mentioning that the different government death registration systems, such as in the various municipalities and states, are not fully connected and that several of them depend on manual work for registration. This can cause discrepancies and delays in data traffic and, consequently, in the production of timely and reliable information. This disconnect between the different systems was observed in our study, by demonstrating the divergence between the Civil Registry Transparency Portal and the InfoGripe.

InfoGripe is a system powered by data from the Influenza Surveillance Information System (SIVEP-Gripe) of the MH and SINAN system for the notification and investigation of cases of diseases and conditions that appear in the National List of Compulsory Notification Diseases (30). COVID-19 was added to this list on February 17, 2020 (31) and, according to the guidelines of the MH, all deaths due to severe acute respiratory syndrome associated with coronavirus (SARS-CoV-2), regardless of hospitalization, must be notified in SIVEP-Gripe, including those occurring in municipalities that do not have a SIVEP-flu record, as they do not have a hospital unit (32). Thus, this notification system is not restricted to the hospital environment and considers deaths at home or in the community. However, it is worth mentioning that, despite this system considering both environments, health and community, in Minas Gerais the pandemic generated an increase in deaths at home due to respiratory causes very similar to COVID-19, such as pneumonia, respiratory failure, sepsis, and SARI, when comparing the months from January to June of the years 2019 and 2020. Thus, given the difficulties in diagnosing and testing the disease, such as performing necropsies and collecting biological materials, it is possible that there would be a rise in underreported deaths by COVID-19 (33).

Furthermore, COVID-19 is closely related to high population density, since it is a contagious disease. In 2012, Minas Gerais occupied the 9th position in the national ranking of urbanization, which corroborates the state's greater vulnerability to the disease. However, in addition to extension, other factors influence vulnerability to the disease, such as education, sanitation, and development indicators (34). Thus, the state of MG has one of the best rates of education, economy, work, and income when compared to other states in the country (22) and that contributes to reduce the state's vulnerability to COVID-19. On the other hand, like most developing countries, Brazil has tested little when compared to developed countries and for the state of MG this reality is no different. The state has the third lowest number of tests per 100,000 inhabitants, a fact that contributed to the failure in the identification of potential transmitters and in counting the number of reported cases, leading to underreporting of the disease, especially when adding possible underreporting for different causes (34).

WHO has been advising countries on the need to expand laboratory testing capacity as a strategy to overcome the pandemic (35). This action will enable the collection of correct information regarding a population's immunity, providing reliable statistics for a better understanding of the circulation of the disease. Consequently, strategies to control the pandemic and even the relaxation of non-pharmacological measures, such as social isolation and quarantine, may be proposed. In Brazil, a network formed by referenced laboratories was established to help fight COVID-19 (19). However, the country is far below the optimal number of tests for COVID-19, as there are not enough tests to display an accurate account of the real number of cases and deaths. This scenario leads to a delay in compiling the records of COVID-19 in Brazil.

It is important to consider that this study can be influenced by limitations related mainly to the source databases consulted. These limitations are inherent to data processing, such as collection, recording, and punctuality, and can cause instability, leaving them subject to change. As a relevant limitation, this article highlights the lack of integration between the two sources consulted, the Civil Registry Transparency Portal and InfoGripe, and the different data collection systems used by them, which could influence the discrepancy between the data presented. The lack of integration between different existing information systems makes it impossible to integrate information from different sources (36). For this reason, several statistical models are proposed to correct this delay, which shows a possible justification for the discrepancy in data observed between the two systems used in the article (37). Therefore, it is necessary to address the methodology used in each database. The Civil Registry Transparency Portal, maintained by ARPEN, has a 14-day delay from death to the updating of its system and InfoGripe monitors the notification data for severe acute respiratory infection (SARI) in Brazil using the SINAN system (24, 38) and the approximate period between notification in SINAN and the data presented on the InfoGripe website is 22 days (39). However, due to the emergency of the pandemic, a technical standard was instituted, and hospitalized SARI cases were immediately notified in SIVEP-Gripe (40). This probably streamlined the process and shortened the deadline, since the registration could be streamlined by eliminating the need for communication between the State Health Secretariats and the SVS. Regarding the system methodology, InfoGripe uses a statistical model that estimates the most recent data, such as the use of the delay pattern between the first symptoms and the date of an update to estimate the cases that have not yet been added. In addition, the model also corrects possible false positives, which could explain the lower SRAG rates presented by the system (38).

Therefore, our results reveal that deaths due to COVID-19 in the state of Minas Gerais may be higher than the official statistics presented. In view of these aspects, it is necessary to expand the diagnostic capacity of Brazil, which will allow us to recognize the real number of deaths and cases of COVID-19 in Minas Gerais. It is also worth mentioning that, although the spatial profile of this study is the state of MG, these observations and analysis can represent the general scenario experienced throughout the country. That is why the data presented here are important, as they reveal the need for other studies to be carried out in order to analyze the real situation of COVID-19 deaths in different Brazilian states; thus, we are able to improve the strategies to contain the COVID-19 pandemic in Brazil.

## Data Availability Statement

The raw data supporting the conclusions of this article will be made available by the authors, without undue reservation.

## Ethics Statement

Ethical review and approval was not required for the study on human participants in accordance with the local legislation and institutional requirements. Written informed consent from the participants' legal guardian/next of kin was not required to participate in this study in accordance with the national legislation and the institutional requirements.

## Author Contributions

SO, TA, and TS contributed to the conception of the study. TA contributed to the acquisition, analysis, interpretation of data, and creation of tables. TS contributed to the statistical analysis, interpretation of data, and creation of tables and figures. NR and SS participated in revising the manuscript critically for important intellectual content within the discussion topic. All the authors co-wrote the paper and give final approval to the version submitted.

## Conflict of Interest

The authors declare that the research was conducted in the absence of any commercial or financial relationships that could be construed as a potential conflict of interest.
